# Commentary: Case report: Chronic neutrophilic leukemia associated with monoclonal gammopathies. A case series and review of genetic characteristics and practical management

**DOI:** 10.3389/fonc.2024.1360791

**Published:** 2024-02-27

**Authors:** Yifan Deng, Shuai Han, Xiaohui Gao, Yang Liu, Jiapei Gao

**Affiliations:** ^1^Yangzhou University Medical College, Yangzhou, Jiangsu, China; ^2^Department of Hematology, The Affiliated Hospital of Yangzhou University, Yangzhou University, Yangzhou, Jiangsu, China

**Keywords:** chronic neutrophilic leukemia, monoclonal gammopathy of undetermined significance, CSF3R, gene mutations, diagnosis, pathogenesis

## Introduction

Chronic neutrophilic leukemia (CNL) is a rare *BCR-ABL*-negative myeloproliferative neoplasm (MPN), characterized by increased peripheral blood mature neutrophils, proliferation of bone marrow mature neutrophils, and hepatosplenomegaly. Most patients with CNL have no obvious symptoms and often seek medical attention due to elevated peripheral blood white blood cells detected during physical examinations. Patients often go through an asymptomatic or mild stage, characterized by elevated neutrophils, with or without splenomegaly, which can last for several months to years, and may be misdiagnosed as infection or tumor causing secondary neutrophilia (1). Due to limitations in understanding of early-stage CNL, most previously reported cases of CNL associated with plasma cell disease (PCD) had reactive neutrophilia, which is a leukemia-like reaction caused by PCD. In 2013, Maxson et al. discovered *CSF3R* mutations in most patients with CNL, and in 2016, the World Health Organization (WHO) updated the diagnostic criteria for CNL, ushering in the era of prospective molecular biology diagnosis for this disease (2, 3).

Vermeersch et al. published a case series of 5 cases of CNL associated with PCD (CNL-PCD) (4), however, 3 cases did not appear to meet the 2016 WHO diagnostic criteria for CNL. We identified 16 cases of CNL associated with PCD (including cases 3 and 4 reported by Vermeersch) that meet the latest WHO diagnostic criteria by searching the PubMed and CNKI databases (**Supplementary Material**). Here, we analyze the clinical characteristics of these 16 cases of CNL-PCD, alongside the findings of Vermeersch et al., with a focus on CNL-PCD diagnosis and molecular biology, as well as common pathogenic features of CNL and PCD.

## Discussion

Compared to previous diagnostic criteria for CNL, the 2016 diagnostic criteria emphasize (2) the presence of *CSF3R* T618I or other activating *CSF3R* mutation; if there is no *CSF3R* mutation, the criteria specify persistent neutrophilia (≥ 3 months), splenomegaly, and no other identifiable cause of reactive neutrophilia, including absence of PCD, or if present, demonstration of clonality of myeloid cells by cytogenetic or molecular studies. In the report of Vermeersch et al., no evidence of myeloid clones was detected using cytogenetic or molecular biology methods in cases 1, 2, and 5, who had increased neutrophils. Therefore, these cases do not meet the strict WHO diagnostic criteria for CNL and may be caused by PCD leading to an increase in reactive neutrophils. Further, the 16 cases of CNL-PCD that we summarize are all proven through genetic testing to have non-reactive elevated white blood cells, meeting the latest diagnostic criteria for CNL-PCD.

In 2017, Clech et al. studied 667 patients with MPN, including 271 with polycythemia vera (PV) and 396 with essential thrombocythemia (ET), and found (5) that 13.9% of patients with MPN carried M protein. In 2020, Javorniczky et al. found (6) that 10 of 114 cases of MPN, including 48 ET, 34 PV, and 32 primary myelofibrosis, carried M protein. These reports suggest that the coexistence of these two different types of disease is not uncommon. As a rare subtype of MPN, CNL has been reported as associated with PCD in 16 cases to date. In addition, we also found two cases of CNL associated with lymphoma, namely follicular lymphoma and diffuse large B-cell lymphoma (4, 7). This phenomenon suggests that CNL may be more inclined to associated with PCD instead of coexisting by chance. The development of MPN is related to chronic inflammation, which stimulates bone marrow production of sufficient numbers of polyclonal granulocytes, monocytes, and macrophages, to ensure destruction of damaged cells and tissues, adequate phagocytosis, and presentation of antigens to lymphocytes (8). Persistent inflammation is associated with an increased risk of cellular DNA alterations in damaged tissues and overstimulated hematopoietic progenitors. Over time, genetic defects are acquired in inflammatory tissues and hematopoietic progenitor cells, ultimately promoting the development of solid cancers or clonal hematopoietic and hematological malignancies. In MPNs, the inflammatory tumor microenvironment both directly promotes progression of clonal myeloproliferation and has important immunosuppressive effects on cytotoxic T cells and other antitumor defenses. In addition, MPN-related driver mutations, including *JAK2* V617F, exert broad pro-inflammatory effects and contribute to maintenance of the malignant inflammatory microenvironment, as well as evasion from T cell immunosurveillance, eventually resulting in uncontrolled clonal escape, providing a potential opportunity for development of B cell tumors (9). In addition, Macauda et al. (10) found that myeloproliferative neoplasms and multiple myeloma share the same genetic susceptibility loci.

Among the 16 cases of CNL-PCD we summarized, 62.5% (10/16) carried *CSF3R* mutations, which is lower than the mutation frequency of *CSF3R* in isolated CNL. As a driving mutation in CNL, *CSF3R* alteration leads to decreased mutation frequencies in both myeloid and lymphoid clones. *CSF3R* mutation in CNL-PCD requires further investigation in more cases. As further mutations were not detected in some of the 16 cases we evaluated, we were unable to accurately calculate the mutation frequencies of additional mutations; however, similar to isolated CNL, we found relatively high mutation frequencies of *SETBP1* and *ASXL1* in CNL-PCD (11). The median mutation frequencies of *ASXL1* and *SETBP1* in isolated CNL are 57.1% and 35.7%, respectively, and these alterations are poor prognostic factors for patients with CNL and CNL-PCD (12). We also found mutations in additional genes, including *JAK2*, *RUNX1*, *NARS*, and *TET2*, among others, in CNL-PCD. The significance of these mutations in CNL-PCD requires support from more samples.

At present, the management for CNL patients include allogeneic hematopoietic stem cell transplantation (allo-HSCT), cytoreductive therapy and targeted drug therapy (13). The first-line treatment of CNL is mainly to reduce the hematological malignancy burden. Hydroxyurea is the most commonly used drug, and 75% of newly diagnosed patients are effective in hydroxyurea treatment (13). In recent years, the targeted drug JAK inhibitor ruxolitinib has become a new direction for CNL treatment, although ruxolitinib treatment achieves short-term hematologic and molecular-biological remission, it does not slow disease progression, and most CNL patients develop drug resistance (12). However, allo-HSCT is the only possible curative treatment. A recent study found that the one-year overall survival rate of CNL patients treated with allo-HSCT was 69%, and the most common cause of death was the primary disease, followed by infection (14). In our summary of 16 cases of CNL-PCD patients, when associated with MGUS, most patients were treated with hydroxyurea and/or ruxolitinib, when associated with MM, the patients were treated with MM-related chemotherapy, we also found one case was recovered after allo-HSCT treatment.

The prognosis of patients with CNL is poor, with a median survival of approximately 24 months (1). Pardanani et al. (7) found that five patients with monoclonal gammopathy of undetermined significance (MGUS) and CNL manifestations did not carry *CSF3R*, *JAK2*, or *SETBP1* mutations. Further, patients with MGUS with neutrophilia had significantly longer survival than those with isolated CNL. Vermeersch et al. (4) found that median survival duration after diagnosis of CNL was 8 years, which may be attributable to some misdiagnoses of CNL; however, median survival of patients in our summary was 22 months, similar to the median survival duration of patients with isolated CNL.

## Conclusion

In summary, according to the 2016 WHO criteria, for diagnosis of CNL associated with PCD, evidence of myeloid clones must be demonstrated through cytogenetic or molecular biology methods. In addition, combined CNL and PCD is very rare, and the reason for coexistence of these two different types of malignancy remains unclear. The chronic inflammatory microenvironment of MPN bone marrow promotes the monoclonal proliferation of B cells. In addition, MPN and multiple myeloma share a common genetic susceptibility site, which may be the mechanism underlying their coexistence. Although the *CSF3R* mutation frequency in CNL-PCD is lower than that in isolated CNL, it is still 62.5%. We believe that future updates and refinement of CNL diagnostic criteria by WHO, as well as the popularization of high-throughput gene detection technologies, such as next generation sequencing, will further deepen our understanding of the molecular biology characteristics of CNL-PCD.

## Author contributions

JG: Writing – review & editing. YD: Writing – original draft. SH: Writing – original draft. XG: Writing – original draft. YL: Writing – original draft.

## References

[1] Thomopoulos TP Symeonidis A Kourakli A Papageorgiou SG Pappa V. Chronic neutrophilic leukemia: A comprehensive review of clinical characteristics, genetic landscape and management. Front Oncol. (2022) 12:891961. doi: 10.3389/fonc.2022.891961 35494007 PMC9048254

[2] Arber DA Orazi A Hasserjian R Thiele J Borowitz MJ Le Beau MM. The 2016 revision to the World Health Organization classification of myeloid neoplasms and acute leukemia. Blood. (2016) 127:2391–405. doi: 10.1182/blood-2016-03-643544 27069254

[3] Maxson JE Gotlib J Pollyea DA Fleischman AG Agarwal A Eide CA. Oncogenic CSF3R mutations in chronic neutrophilic leukemia and atypical CML. N Engl J Med. (2013) 368:1781–90. doi: 10.1056/NEJMoa1214514 PMC3730275 23656643

[4] Vermeersch G Delforge M Havelange V Graux C Michaux L Devos T. Case report: Chronic neutrophilic leukemia associated with monoclonal gammopathies. A case series and review of genetic characteristics and practical management. Front Oncol. (2022) 12:1014671. doi: 10.3389/fonc.2022.1014671 36568246 PMC9768602

[5] Le Clech L Sakka M Meskar A Kerspern H Eveillard JR Berthou C. The presence of monoclonal gammopathy in Ph-negative myeloproliferative neoplasms is associated with a detrimental effect on outcomes. Leuk Lymphoma. (2017) 58:2582–7. doi: 10.1080/10428194.2017.1312380 28482711

[6] Javorniczky NR Wehrle J Ihorst G Hupfer V Aumann K Pfeifer D. Prevalence and characteristics of myeloproliferative neoplasms with concomitant monoclonal gammopathy. Leuk Res. (2020) 98:106454. doi: 10.1016/j.leukres.2020.106454 32971364

[7] Pardanani A Lasho TL Laborde RR Elliott M Hanson CA Knudson RA. CSF3R T618I is a highly prevalent and specific mutation in chronic neutrophilic leukemia. Leukemia. (2013) 27:1870–3. doi: 10.1038/leu.2013.122 PMC4100617 23604229

[8] Hermouet S Bigot-Corbel E Gardie B. Pathogenesis of myeloproliferative neoplasms: role and mechanisms of chronic inflammation. Mediators Inflamm. (2015) 2015:145293. doi: 10.1155/2015/145293 26538820 PMC4619950

[9] Nasillo V Riva G Paolini A Forghieri F Roncati L Lusenti B. Inflammatory microenvironment and specific T cells in myeloproliferative neoplasms: immunopathogenesis and novel immunotherapies. Int J Mol Sci. (2021) 22. doi: 10.3390/ijms22041906 PMC7918142 33672997

[10] Macauda A Giaccherini M Sainz J Gemignani F Sgherza N Sanchez-Maldonado JM. Do myeloproliferative neoplasms and multiple myeloma share the same genetic susceptibility loci? Int J Cancer. (2021) 148:1616–24. doi: 10.1002/ijc.33337 33038278

[11] Gao JP Zhai LJ Gao XH Min FL. Chronic neutrophilic leukemia complicated with monoclonal gammopathy of undetermined significance: A case report and literature review. J Clin Lab Anal. (2022) 36:e24287. doi: 10.1002/jcla.24287 35170077 PMC8993655

[12] Gao J Han S Deng B Deng Y Gao X. Research progress of additional pathogenic mutations in chronic neutrophilic leukemia. Ann Hematol. (2023). doi: 10.1007/s00277-023-05550-6 37993585

[13] Szuber N Tefferi A. Current management of chronic neutrophilic leukemia. Curr Treat Options Oncol. (2021) 22:59. doi: 10.1007/s11864-021-00856-x 34097138

[14] Dholaria B Radujkovic A Estrada-Merly N Sirait T Kim S Hernandez-Boluda JC. Outcomes of allogeneic haematopoietic cell transplantation for chronic neutrophilic leukaemia: A combined CIBMTR/CMWP of EBMT analysis. Br J Haematol. (2022) 198:785–9. doi: 10.1111/bjh.18297 PMC9750039 35658101

