# Supplementary material for: Commentary: Case report: Chronic neutrophilic leukemia associated with monoclonal gammopathies. A case series and review of genetic characteristics and practical management
Source: Front Oncol. 2024 Feb 27;14:1360791. doi: 10.3389/fonc.2024.1360791 (PMC10928800; doi:10.3389/fonc.2024.1360791)
Supplement: Supplementary file 1 [file Table_1.docx]

| **Number** | **Published years, study and reference** | **Age(year)/Sex** | **Types of PCD** | **Gene mutations** | **Management** | **Status and Survival (months)** |
| --- | --- | --- | --- | --- | --- | --- |
| 1 | Nedeljkovic et al. 2014(1) | 81/M | IgG-κ type SMM | *JAK2* V617F | - | *-* |
| 2 | Blombery et al. 2014(2) | 57/F | IgG-κ type MGUS | *CSF3R* T618I  *CSF3R* S783fs | - | *-* |
| 3 | Stevens et al. 2016(3) | 69/M | IgA-κ type MGUS | *SETBP1* | Hu | Dead; 11 |
| 4 |  | 70/M | MGUS | *SETBP1* | Hu | Dead; 22 |
| 5 | Jiang et al. 2016(4) | 58/M | IgA-κ type MM | *CSF3R* P733T | TCD | Alive; 89 |
| 6 | Fathi et al. 2017(5) | 86/F | MGUS | *CSF3R* T618I, *TET2*,  *ASXL1*, *SETBP1*, *TP53* | Hu, Ruxolitinib | Dead; 17 |
| 7 | Bredeweg et al. 2018(6) | 53/M | MGUS | *CSF3R*, *SETBP1* | Ruxolitinib | - |
| 8 | He et al. 2019(7) | 87/M | IgA-λ type MM | *CSF3R*, *ASXL1* | - | Dead; 4 |
| 9 | Ge et al. 2020(8) | 77/M | IgD-λ type MM | *CSF3R* T618I | Hu | Alive; 3 |
| 10 | Han et al. 2020(9) | 73/M | IgG-κ type MGUS | *ASXL1*, *NRAS* | - | - |
| 11 | Hobbs et al. 2021(10) | 72/M | MM | *CSF3R* T618I, *SF3B1* | - | - |
| 12 | Gao et al. 2022(11) | 73/M | IgG-λ type MGUS | *CSF3R* T618I, *ASXL1*, *RUNX1* | Hu, Ruxolitinib | Dead; 34 |
| 13 | Lauw et al. 2022(12) | 88/F | IgG-λ type MGUS | *JAK2* V617F, *ASXL1* | - | Dead; 1 |
| 14 | McVinnie et al. 2022(13) | 85/F | IgG-λ type MM | *CSF3R* T618I  *CSF3R* S810fs | RCD | Alive; 38 |
| 15 | Vermeersch et al. 2022(14) | 73/F | λ light chain MM | *ASXL1* | VMP | Alive; 48 |
| 16 |  | 69/M | IgG-λ type MGUS | *CSF3R* T618I, *SETBP1*,  *U2AF1* | Decitabine  Allo-HSCT | Alive; 24 |

**Clinical features of 16 CNL-PCD patients**

**Abbreviation**: CNL-PCD: chronic neutrophilic leukemia complicated with plasma cell disorder; PCD: plasma cell disorder; SMM: smoldering multiple myeloma; MGUS: monoclonal gammopathy of undetermined significance; MM: multiple myeloma; -: not given; Hu: hydroxyurea;

TCD: Thalidomide-cyclophosphamide-dexamethasone; RCD: Lenalidomide- cyclophosphamide-dexamethasone; VMP: Bortezomib-melphalan-prednisone; Allo-HSCT: Allogeneic stem cell transplantation

1. Nedeljkovic M, He S, Szer J, Juneja S. Chronic neutrophilia associated with myeloma: is it clonal? Leuk Lymphoma. 2014;55(2):439-40.

2. Blombery P, Kothari J, Yong K, Allen C, Gale RE, Khwaja A. Plasma cell neoplasm associated chronic neutrophilic leukemia with membrane proximal and truncating CSF3R mutations. Leuk Lymphoma. 2014;55(7):1661-2.

3. Stevens B, Maxson J, Tyner J, Smith CA, Gutman JA, Robinson W, et al. Clonality of neutrophilia associated with plasma cell neoplasms: report of a SETBP1 mutation and analysis of a single institution series. Leuk Lymphoma. 2016;57(4):927-34.

4. Jiang B, Qi JY, Li QH, Xu Y, Sun MY, Zheng WW, et al. [Chronic neutrophilic leukemia complicated with multiple myeloma: two cases report and literature review]. Zhonghua Xue Ye Xue Za Zhi. 2016;37(8):688-91.

5. Fathi AT, Graubert TA, Kulkarni NM, Kuo FC, Hasserjian RP. Case 37-2016. An 86-Year-Old Woman with Leukocytosis and Splenomegaly. N Engl J Med. 2016;375(23):2273-82.

6. Bredeweg A, Burch M, Krause JR. Chronic neutrophilic leukemia. Proc (Bayl Univ Med Cent). 2018;31(1):88-9.

7. He M, Zhao XC, Bai H. [Chronic neutrophilic leukemia with CSF3R mutation and concurrent multiple myeloma: one case report]. Zhonghua Xue Ye Xue Za Zhi. 2019;40(12):1052.

8. Ge GX, Feng HY, Fu LH. IgD-lambda multiple myeloma complicated with chronic neutrophilic leukemia: a case report. Linchuang Xue Ye Xue Za Zhi. 2020;33(09):647-649.

9. Han Q, Tao L, Jiang X, Chen H, Liu C, Sun M. An atypical chronic neutrophilic leukemia patient harboring ASXL1 and NRAS mutations associated with monoclonal plasma cell dyscrasia presenting as hemopericardium. Ann Hematol. 2021;100(1):273-5.

10. Hobbs LK, Carr PC, Gru AA, Flowers RH. Case and review: Cutaneous involvement by chronic neutrophilic leukemia vs Sweet syndrome- A diagnostic dilemma. J Cutan Pathol. 2021;48(5):644-9.

11. Gao JP, Zhai LJ, Gao XH, Min FL. Chronic neutrophilic leukemia complicated with monoclonal gammopathy of undetermined significance: A case report and literature review. J Clin Lab Anal. 2022;36(4):e24287.

12. Lauw MIS, Hakim N, Arora S, Prakash S, Xie Y. A case of plasma cell neoplasm-associated chronic neutrophilic leukemia with ASXL1 and JAK2V617F mutations. Ann Hematol. 2022;101(8):1879-81.

13. McVinnie K, Innes A, Nadal-Melsio E, Atta M, Deplano S. A case of chronic neutrophilic leukemia and multiple myeloma showing the benefits of lenalidomide and cyclophosphamide therapy in treating both conditions. Am J Hematol. 2022;97(11):1491-4.

14. Vermeersch G, Delforge M, Havelange V, Graux C, Michaux L, Devos T. Case report: Chronic neutrophilic leukemia associated with monoclonal gammopathies. A case series and review of genetic characteristics and practical management. Front Oncol. 2022;12:1014671.
